# Publishing Trends of Editorial Board Members in Top Dermatology Journals

**DOI:** 10.7759/cureus.50518

**Published:** 2023-12-14

**Authors:** Matthew H Friedland, Mickey Nguyen, Yolanda R Helfrich

**Affiliations:** 1 Dermatology, University of Michigan, Ann Arbor, USA

**Keywords:** authorship, risk of bias, transparency, editorial board member, publication trends

## Abstract

Introduction

While some medical specialties have examined the effect of editorial board membership on the likelihood of manuscript publication, this has been minimally studied in dermatology. We investigated the publication patterns of 67 editorial board members at three leading dermatology journals to identify any discernible patterns between editorial board membership and publication rates.

Materials and methods

Using Scopus, Elsevier’s author search tool, we identified editorial board members who served continuously over a three-year period between January 2019 and December 2021 at JAMA Dermatology (JAMA Derm), the British Journal of Dermatology (BJD), and the Journal of the Academy of Dermatology (JAAD). All data are from publicly available sources.

Results

The mean difference in the number of publications within a member’s own journal compared to those published in the other top two journals was significantly higher for JAAD (8.6 [95% CI 2.0 to 15.2]; P = 0.013) and BJD (4.3 [95% CI, 2.3 to 6.2]; P = 1.4E-05), but not for JAMA Derm (-3.8 [95% CI, -1.53 to 9.0]; P= 0.07). The mean difference in the percent of total publications appearing in a member’s own journal compared to the percent appearing in the other top two journals was significantly higher for JAAD (30.5% [95% CI, 17% to 44%]; P = 0.00016) and BJD (17.0% [95% CI, 9.2% to 24.7%]; P = 6.7E-05), but not for JAMA Derm (-6.3% [95% CI, -15.7% to 3.1%]; P = 0.18).

Discussion

Although we make no claims about irregular practices, the role of editorial board members as “gatekeepers” of publication can lead to allegations of potential bias, favoritism, and conflicts of interest. The high proportion of in-journal publications for editorial board members of JAAD and BJD is, therefore, worth further consideration.

Conclusion

These results may indicate that reflection on the manuscript review and publication process is warranted to ensure equity and inclusivity. Some limitations of this study include the short time interval of three years, the inclusion of only three journals, and the lack of established causation. Further examination of editorial review and publication practices should be undertaken.

## Introduction

Publishing papers in academic journals is a cornerstone of many physician careers. From sharing groundbreaking scientific discoveries to advancing professional aspirations, dermatologists and other physicians contribute to scientific progress through publications. Editors and editorial boards serve as the gatekeepers of medical literature; through scientific review, they determine which articles are published and which are not. Most editors are also active scientific researchers creating their own academic work and submitting it for publication. A potential conflict of interest can arise when an editor or editorial board member submits their work to their own journal, even if other editors are reviewing the work. Some journals publish work by their own editors; others do not [1]. Some journals may encourage editors and board members to submit their work to their own journals. While some medical specialties have examined the effect of editorial board membership on the likelihood of manuscript publication, this has been minimally studied in dermatology [1-5]. Within some journals, a small number of authors, frequently editorial board members, have been found to have a disproportionately high number of publications in their own journal, as well as shorter wait times for manuscript processing and publication decisions [6,7]. We investigated the publication patterns of 67 editorial board members at three leading dermatology journals to identify patterns between editorial board membership and publication rates and compare publication rates within their own journals versus external journals.

## Materials and methods

Using Scopus, Elsevier’s author search tool, we identified editorial board members who served continuously between January 2019 and December 2021 at JAMA Dermatology (JAMA Derm), the British Journal of Dermatology (BJD), and the Journal of the Academy of Dermatology (JAAD). These journals have consistently ranked in the top five dermatology journals by impact factor over the last five years, according to Clarivate Web of Science citation reports [8]. We considered editorial board members with titles of Associate Editor, Assistant Section Editor, and higher, and with more than six total publications over the period studied. Editorial staff members who were not dermatologists and publishing staff were excluded. Editorial articles were also excluded due to their regular publication cadence. Initial data were collected from 104 authors, with 67 authors in the final analysis based on exclusion criteria. All data were collected from publicly available sources, and no humans were contacted during any part of the research process. Data were collected on each author’s h-index, editorial board title, number/type of articles in their own journal, number/type of articles in other top two journals, total publications, and affiliations between January 2019 and December 2021. Average and median publications were calculated; t-tests were run to examine differences in publication rates and differences in the percentage of total publications in their own journal. P-values <0.05 were considered significant.

## Results

For the 67 authors included, the average percentages of members’ total publications (excluding editorials) appearing within their own journal were 13.4% (JAMA Derm), 23.6% (BJD), and 35.0% (JAAD). The average percentages of a member’s total publications appearing in the other top two journals were 19.8% (JAMA Derm), 6.6% (BJD), and 4.56% (JAAD). The percentages of total publications within a member’s own journal as compared to the number of publications in all three top journals were 42.4% (JAMA Derm), 78.3% (BJD), and 82.8% (JAAD) (Table 1).

**Table 1:** Descriptive statistics of authors (January 2019 to December 2022)

Descriptive Statistics	JAMA Derm	BJD	JAAD	Average	Total
Number of Included Authors	16	34	17	22.3	67
Total Publications without Editorials	665	1,411	528	868.0	2,604
Publications in Editorial Member’s Own Journal without Editorials	68	233	173	158.0	474
Total Publications in JAMA, BJD, & JAAD without Editorials	196	321	200	239.0	717
Average h-index	30.1	33.0	21.4	28.1	–
Percentages	JAMA Derm	BJD	JAAD	Average	
Percent of Total Publications Appearing in Members’ Own Journal	13.4%	23.6%	35.0%	24.0%	–
Percent of Total Publications Appearing in other Top Two Journals of which Member is not on the Editorial Board	19.8%	6.6%	4.56%	10.3%	–
Percent of Publications in JAMA, BJD, & JAAD Appearing in Members’ Own Journal	42.4%	78.3%	82.8%	67.8%	–

Within the examined period, the mean difference in the number of publications within a member’s own journal compared to those published in the other top two journals was significantly higher for JAAD (8.6 [95% CI 2.0 to 15.2]; P = 0.013) and BJD (4.3 [95% CI, 2.3 to 6.2]; P = 1.4E-05), but not for JAMA Derm (-3.8 [95% CI, -1.53 to 9.0]; P = 0.07). The mean difference in the percent of total publications appearing in a member’s own journal compared to the percent appearing in the other top two journals was significantly higher for JAAD (30.5% [95% CI, 17% to 44%]; P = 0.00016) and BJD (17.0% [95% CI, 9.2% to 24.7%]; P = 6.7E-05), but not for JAMA Derm (-6.3% [95% CI, -15.7% to 3.1%]; P = 0.18) (Table 2).

**Table 2:** Results of t-tests *Statistically significant if p < 0.05

Statistical Analysis	JAMA Derm	BJD	JAAD
Mean Difference in Publications in Member’s Own Journal compared to Publications in other Top Two Journals	-3.8	4.3*	8.6*
	[95% CI -1.53 to 9.0]	[95% CI 2.3 to 6.2]	[95% CI 2.0 to 15.2]
	P = 0.07	P = 1.4E-05	P = 0.013
Mean Difference in Percent of Total Publications Appearing in Member’s Journal compared to Percent of Total Publications Appearing in other Top Two Journals	-6.3%	17.0%*	30.5%*
	[95% CI -15.7% to 3.1%]	[95% CI 9.2% to 24.7%]	[95% CI 17% to 44%]
	P = 0.18	P = 6.7E-05	P = 0.00016

## Discussion

While it may not be surprising to see higher publication rates for editorial board members within their own journals, this has not yet been well-studied in dermatology [1-5]. A large systematic review examining the phenomenon of “self-publishing” found considerable variability across different fields, journals, and editors [1]. Some editors never publish in their own journals, while others publish extensively in their own journals [1]. Editorial board members often have a strong track record of publication within a field. They are frequently regarded as key thought leaders, have high h-indexes, and may be more aware of journal priorities and trending topics. All of these factors may contribute to a higher likelihood that their papers are accepted for publication. However, the role of editorial board members as “gatekeepers” of publication can raise concerns about potential bias, favoritism, and conflicts of interest [2].

It is important to be clear that we make no claims about bias or irregular practices within the three journals examined in this article. Nevertheless, the high proportion of in-journal publications for editorial board members is worth further consideration. The pressure to publish seems to intensify every year, yet the ability to publish is increasingly challenging for many. As dermatology strives to prioritize diversity, equity, and inclusion, this emphasis should extend to manuscript review and publication, ensuring that a variety of voices is being sought out and heard. It is crucial to balance the voices of the influential with fresh perspectives.

To address concerns of preferential treatment or bias, Helgesson et al. propose that journals be transparent about the criteria employed in the review process and the review of editorial board member submissions to the journal. They suggest that journals exclude editors from any formal influence over the review and acceptance of their own submissions [1]. Additionally, they advocate for blinding the identities of editorial board members from reviewers as part of the manuscript review process. Helgesson et al. further recommend that editors-in-chief, and perhaps associate editors, should avoid publishing within their own journals [1].

## Conclusions

The findings of this study may suggest that further reflection on the manuscript review process is warranted. Increasing transparency about the factors considered during the review process for all submissions, especially those by editorial board members, could alleviate concerns about potential favoritism or bias. It is important not to assume improper practices from these data but rather use it as an opportunity to conduct meaningful introspection of journal review practices as we strive to eliminate bias in publication. Having a small number of authors with a disproportionately high number of publications over a long period of time should prompt a review to ensure that journals include a diverse set of voices. This study is limited by its short time frame of three years, its inclusion of only three journals, and its inability to establish causation. Further examination of editorial review and publication practices should be conducted with a larger cohort of journals over a more extended period of time. A comparison of publication rates before and after becoming an editorial board member may also be useful.
